# Bat optimization of hybrid neural network-FOPID controllers for robust robot manipulator control

**DOI:** 10.3389/frobt.2025.1487844

**Published:** 2025-05-02

**Authors:** Bashra Kadhim Oleiwi, Mohamed Jasim, Ahmad Taher Azar, Saim Ahmed, Ahmed Redha Mahlous

**Affiliations:** ^1^ Department of Control and System Engineering, University of Technology, Baghdad, Iraq; ^2^ College of Computer and Information Sciences, Prince Sultan University, Riyadh, Saudi Arabia; ^3^ Automated Systems and Computing Lab (ASCL), Prince Sultan University, Riyadh, Saudi Arabia

**Keywords:** trajectory tracking, neural network, neural network controller, PIPD controller, PID controller, FOPID controller, bat optimization algorithm, 3-link rigid robotic manipulator

## Abstract

The position and trajectory tracking control of rigid-link robot manipulators suffers from problems such as poor accuracy, unstable performance, and response caused by unidentified loads and outside disturbances. In this paper, three control structures have been proposed to control a multi-input, multi-output coupled nonlinear three-link rigid robot manipulator (3-LRRM) system and effectively solve the signal chattering in the control signal. To overcome these problems, three hybrid control structures based on combinations between the benefits of fractional order proportional-integral-derivative operations (FOPID) and the benefits of neural networks are proposed for a 3-LRRM. The first hybrid control scheme is a neural network- (NN) like fractional order proportional-integral plus an NN-like fractional order proportional derivative controller (NN-FOPIPD) and the second control scheme is an NN plus FOPID controller (NN + FOPID). In contrast, the third control scheme is the Elman NN-like FOPID controller (ELNN-FOPID). The bat optimization algorithm (BOA) is applied to find the best parameter values of the proposed control scheme by minimizing the performance index of the integral time square error (ITSE). MATLAB software is used to carry out the simulation results. Using the simulation tests, the performance of the suggested controllers is compared without retraining the controller parameters. The robustness of the designed control schemes’ performance is assessed utilizing uncertainties in system parameters, outside disturbances, and initial position changes. The results show that the NN-FOPIPD structure demonstrated the best performance among the suggested controllers.

## 1 Introduction

One of the most promising technologies is robots, which are extensively employed in numerous applications in industries like drilling, medical procedures (Chen et al., 2017), transportation (Wu et al., 2016), assembly (Fani et al., 2018), and manufacturing (Sadrfaridpour et al., 2016). The three main categories of robotic manipulator research are robot manipulator control, optimal trajectory planning for industrial manipulators (Ayten et al., 2016), and robotic system design (Zemiti et al., 2007; Ayten et al., 2017). Among these fields of study, robotic manipulator control plays an important and vital role in tracking a reliable and accurate path to the starting and finishing points of the work.

Robotic systems are time-varying nonlinear systems because of external disturbances, modeling uncertainties, and the nonlinear behavior of the system dynamics. Therefore, obtaining robotic systems that track specified paths successfully and accurately is challenging. Many sophisticated control techniques have been developed to overcome these undesirable uncertainties for various types of robotic systems (Tutsoy et al., 2023). These techniques include fractional-order control (Viola and Angel, 2015; Dhivakaran et al., 2022; Kaushik et al., 2024), H-infinity control (Makarov et al., 2016), adaptive control (Huang, 2016; Tutsoy et al., 2024), neural network control (Sun et al., 2017; Rajchakit et al., 2019), and sliding mode control (SMC) (Dumlu et al., 2017; Basci et al., 2017).

Using a fractional-order scheme, several methods have been integrated with other notable schemes to improve the performance. The fractional-order SMC (FOSMC) was proposed by Dumlu (2018) to illustrate a robotic manipulator’s ability to track position practically. Ren et al. (2023) used a unique composite position predictive control method that combines disturbance preview and motion profile techniques to achieve accurate and smooth trajectory regulation. Xia et al. (2019) proposed a control strategy combining the modified terminal sliding mode with the double power reaching law to precisely and quickly track tasks of rigid robotic manipulators. Using fractional calculus, Ullah et al. (2015) proposed a new fuzzy FOSMC for a servo actuation system (). The results showed that the suggested method’s tracking error is less than that of a traditional SMC. For the doubly fed induction generator, Ebrahimkhani (2016) developed an innovative, reliable FOSMC with a fractional-order estimator. The outcomes have validated the suggested controller’s robustness and efficacy in dealing with the effects of changing disturbances and parameters. Andualem and Gebremichael (2020) suggested using a hyperbolic tangent function instead of a signum function in a fuzzy gain scheduling terminal SMC for controlling and tracking the UR5 robot manipulator.

Recently, several research papers have proposed control structures and hybrid controllers for a 2-LRRM that deals with the path-tracking problem. Kumar et al. (2019) suggested a self-regulated FOFPID controller with a backtracking search algorithm. Mohamed et al. (2023) suggested six control structures as a hybrid controller for a 2-LRRM that addresses the trajectory tracking problem by combining the advantages of PID controllers with integer and fractional orders, neural networks, and the Gorilla Forces Troops Optimization algorithm. Six neural network-based control structures with PID and fractional-order PID (FOPID) controllers were proposed by Mohamed et al. (2024) for operating a 2-LRRM for trajectory tracking. These controllers are referred to as NN + PID, NN + FOPID, recurrent neural network-like PID, set-point-weighted FOPID, set-point-weighted PID, and RNN-like FOPID. The parameters of the suggested controllers’ conditions, disturbances, and model uncertainties were modified using the Zebra Optimization algorithm. Three control structures for 3-LRRM were proposed by Mohamed et al. (2023) utilizing a neural network in conjunction with PID actions. The Coot Optimization algorithm is used to modify the parameters of the suggested controllers. Based on the social behavior of spider monkeys, Spider Monkey Optimization (SMO) is another optimization technique used to control robotic manipulators (Abualigah et al., 2024; Agrawal et al., 2023). The PID controller adjusts with SMO to find the best control parameters in the robotic manipulator in order to enhance the manipulator version (Agrawal et al., 2021). Wang et al. (2020) proposed an adaptive PID control algorithm for multi-degree-of-freedom (DOF) industrial robots, leveraging a fuzzy neural network framework to enhance trajectory tracking performance. Their research addresses the complexities inherent in industrial robot control systems, characterized by nonlinearity, time-varying dynamics, and strong coupling effects. By establishing a functional relationship between control error and reaching degree, the authors achieved self-adaptive adjustments of PID parameters, significantly improving control accuracy and stability compared to traditional PID methods. The effectiveness of their approach was validated through simulations and joint experiments using the ADAMS virtual simulation system, demonstrating superior performance in trajectory tracking tasks. In the study conducted by Sau (2020), a comparative analysis of fractional order controllers was performed specifically for a three-link robotic manipulator system. The research highlights the significance of fractional order control strategies in enhancing the dynamic performance and stability of robotic systems. By employing fractional calculus, the study demonstrates that these controllers can provide improved tuning flexibility compared to traditional integer-order controllers. This flexibility allows for more precise adjustments to the system's response characteristics, which is crucial in applications requiring high precision and reliability.

It is evident that each of the methods discussed above has advantages and disadvantages in the majority of studies. They were employed at the expense of accuracy and time due to their complexity and time-consuming computations. Previous investigations indicated that most of the studies did not resolve the signal chattering in the control signal and addressed the suggested controllers separately. In this paper, three control structures have been proposed to control a multi-input, multi-output coupled, nonlinear 3-LRRM system and effectively solve the signal chattering in the control signal. It is well known that the FOPID controller is more robust and flexible than the conventional PID controller. On the other hand, the neural network has high flexibility in mapping complex data. Hence, a hybrid controller consisting of FOPID operations and a neural network will perform better than the FOPID controller alone.

The aim of this work is to design hybrid controllers combining the benefits of FOPID operations and the benefits of neural networks-based BOA for 3-LRRM by solving the nonlinear problems of compensating robot manipulator control with disturbances and uncertainties and achieving precise trajectory tracking.

This work’s primary contributions are as follows:

1 Develop three structures of hybrid control based on combinations between the benefits of FOPID operations and the benefits of neural networks. These hybrid control structures are neural network-like FOPIPD controllers (NN-FOPIPDs), neural network plus FOPID controllers (NN + FOPIDs), and Elman neural network-like FOPID controllers (ELNN-FOPIDs).
2 Apply the bat optimization algorithm (BOA) to determine the optimal parameters for each suggested controller in order to further improve the performance.
3 Conduct a comparative analysis among the suggested controllers concerning changing the starting conditions, outside disruptions, parameter uncertainty, and all combined effects.
4 A new objective function suggestion to fine-tune the suggested controller to generate the control signal with a minimum amount of chattering.

The remaining sections of this work are organized as follows: Section 2 explains the 3-LRRM’s dynamical system. Section 3 illustrates the suggested controllers. Section 4 displays the BOA, and Section 5 displays the simulation’s results. The robustness test is provided in Section 6. The conclusion is given in Section 7.

## 2 System modeling formulation

**FIGURE 1:**
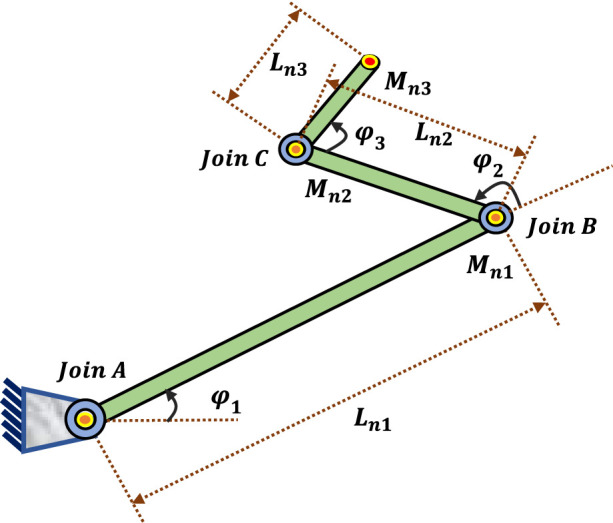
The 3-LRRM structure.

A robotic manipulator is constructed of several links joined by joints at each end. Planar robotic manipulators can only move in one plane (Abdulameer and Mohamed, 2022). This research considers three rotating joints on a planar robotic manipulator, and it is assumed that each joint is actuated (Kumar et al., 2019). Figure 1 shows the 3-LRRM structure. The manipulator’s dynamic motion equation is used to develop the fundamental control equations for robots. The torques produced by actuators in a robotic system cause the dynamic movement of the manipulator arms (Raafat and Raheem, 2017). The following is the dynamic model of a 3-LRRM.

The 3-LRRM’s dynamic model is defined by Lagrange dynamics (Lewis et al., 2004). Equations 1–6 are the equations for the x and y positions of links 1, 2, and 3:

$$x_{1}={\;L}_{n1}\;\cos\;\left(\varphi_{1}\right),$$ (1)

$$y_{1}=L_{n1}\;\sin\;\left(\varphi_{1}\right),$$ (2)

$$x_{2}=L_{n1}\;\cos\;\left(\varphi_{1}\right)+L_{n2}\;\cos\;\left(\varphi_{1}+\varphi_{2}\right),$$ (3)

$$y_{2}=L_{n1}\;\sin\;\left(\varphi_{1}\right)+L_{n2}\;\sin\;\left(\varphi_{1}+\varphi_{2}\right),$$ (4)

$$x_{3}=L_{n1}\;\cos\;\left(\varphi_{1}\right)+L_{n2}\;\cos\;\left(\varphi_{1}+\varphi_{2}\right)+L_{n3}\cos\left(\varphi_{1}+\varphi_{2}+\varphi_{3}\right),$$ (5)

$$y_{3}=L_{n1}\;\sin\;\left(\varphi_{1}\right)+L_{n2}\;\sin\;\left(\varphi_{1}+\varphi_{2}\right)+L_{n3}\sin\left(\varphi_{1}+\varphi_{2}+\varphi_{3}\right).$$ (6)

Kinetic energy (*KinE*) is indicated in Equation 7:

$$KinE=\frac{1}{2}\;M_{n1\;}v_{1}^{2}+\frac{1}{2}\;M_{n2\;}v_{2}^{2}+\frac{1}{2}\;M_{n3\;}v_{3.}^{2}$$ (7)

where $L_{n1}$ , $L_{n2}$ , and $L_{n3}$ and $M_{n1\;}$ , $M_{n2}$ and $M_{n3}$ are the lengths and masses of the links. $\varphi_{1}$ , $\varphi_{2}$ , $\varphi_{3}$ and $v_{1}$ , $v_{2}$ , $v_{3\;}$ represent the positions and velocities of the links, respectively. The velocities can be written as Equation 8.

$$v_{1}={\left({\dot{x}}_{1}^{2}+{\dot{y}}_{1}^{2}\right)}^{0.5},\;v_{2}={\left({\dot{x}}_{2}^{2}+{\dot{y}}_{2}^{2}\right)}^{0.5},\;v_{3}={\left({\dot{x}}_{3}^{2}+{\dot{y}}_{3}^{2}\right)}^{0.5}$$ (8)

The kinetic energy (*KinE)* is presented in Equation 9:

$$KinE=\frac{1}{2}M_{n1}\left({\dot{x}}_{1}^{2}+{\dot{y}}_{1}^{2}\right)+\frac{1}{2}\;M_{n2}\;\left({\dot{x}}_{2}^{2}+{\dot{y}}_{2}^{2}\right)+\frac{1}{2}\;M_{n3}\;\left({\dot{x}}_{3}^{2}+{\dot{y}}_{3}^{2}\right).$$ (9)

The potential energy ( $\left.PotE\right)$ is defined in Equations 10, 11:

$$PotE=\sum_{i=1}^{3}M_{ni\;}g\;h_{i}\left(\varphi \right),$$ (10)

where g represents the acceleration and $h_{i}\left(\varphi \right)$ represents the height.

$$\begin{array}{ll} PotE & =M_{n1}\;gL_{n1}\sin\left(\varphi_{1}\right)+M_{n2}\;g\left(L_{n1\;}\sin\left(\varphi_{1}\right)+L_{n2}\sin\left(\varphi_{1}+\varphi_{2}\right)\right)\\ & \;+\;M_{n3}\;g\left(\;L_{n1}\sin\left(\varphi_{1}\right)+L_{n2}\sin\left(\varphi_{1}+\varphi_{2}\right)+L_{n3}\sin\left(\varphi_{1}+\varphi_{2}+\varphi_{3}\right)\right) \end{array}$$ (11)

Next, the Lagrangian (*L*) was determined using the Lagrange dynamic, as shown in Equation 12:

L=KinE−PotE. (12)

The Euler–Lagrange equation is expressed by Equation 13:

$$\frac{d}{dt}\;\left[\frac{\partial L}{\partial \dot{\varphi_{i}}}\right]-\frac{\partial L}{\partial \varphi_{i}}=F\varphi_{i,}$$ (13)

where $F\varphi_{i}$ represents the torque exerted on link *i*.

The basic principle dynamics of the manipulator are illustrated in Equations 14–24 (Lewis et al., 2004):

$$Q\left(\varphi \right)\ddot{\varphi }+P_{c}\left(\varphi ,{\dot{\varphi }}^{2}\right)+R\left(\varphi ,{\dot{\varphi }}_{i}{\dot{\varphi }}_{j}\right)+G\left(\varphi \right)=\tau ,$$ (14)

where the inertia matrix is denoted by $Q\left(\varphi \right),\text{centrifugal}\;\text{term}$ $P_{c},and\;\text{Coriolis}\;\text{term}\;R$ .

$$Q=\left[\begin{array}{ccc} Q_{11} & Q_{12} & Q_{13}\\ Q_{21} & Q_{22} & Q_{23}\\ Q_{31} & Q_{32} & Q_{33} \end{array}\right],$$ (15)

$$\begin{array}{ll} Q_{11} & =\left(M_{n1}+M_{n2}+M_{n3}\right)L_{n1}^{2}+\left(M_{n2}+M_{n3}\right)L_{n2}^{2}+M_{n3}L_{n3}^{2}\\ & \;\left.+\;2M_{n3}L_{n1}L_{n3\;}\cos(\varphi_{2}+\varphi_{3}\right]+2\left(M_{n2}+M_{n3}\right)L_{n1}L_{n2}\cos\left(\varphi_{2}\right)\\ & \;+2M_{n3}L_{n2}L_{n3}\cos\left(\varphi_{3}\right) \end{array}$$ (16)

$$\begin{array}{ll} Q_{12} & =\left(M_{n2}+M_{n3}\right)L_{n2}^{2}+M_{n3}L_{n3}^{2}+M_{n3}L_{n1}L_{n3}\cos\left(\varphi_{2}+\varphi_{3}\right)\\ & \;+\left(M_{n2}+M_{n3}\right)L_{n1}L_{n2}\cos\left(\varphi_{2}\right)+2M_{n3}L_{n2}L_{n3}\cos\left(\varphi_{3}\right), \end{array}$$ (17)

$$Q_{13}=M_{n3}L_{n3}^{2}+M_{n3}L_{n1}L_{n3}\cos\left(\varphi_{2}+\varphi_{3}\right)+M_{n3}L_{n2}L_{n3}\cos\left(\varphi_{3}\right),$$ (18)

$$\begin{array}{ll} Q_{21} & =M_{n2}L_{n2}^{2}+M_{n3}L_{n2}^{2}+M_{n3}L_{n3}^{2}+M_{n3}L_{n1}L_{n3}\cos\left(\varphi_{2}+\varphi_{3}\right)\\ & \;+M_{n2}L_{n1}L_{n2}\cos\left(\varphi_{2}\right)+M_{n3}L_{n1}L_{n2}\cos\left(\varphi_{2}\right)\\ & \;+2M_{n3}L_{n2}L_{n3}\cos\left(\varphi_{3}\right), \end{array}$$ (19)

$$Q_{22}=M_{n2}L_{n2}^{2}+M_{n3}L_{n2}^{2}+M_{n3}L_{n3}^{2}+2M_{n3}L_{n2}L_{n3}\cos\left(\varphi_{3}\right),$$ (20)

$$Q_{23}=M_{n3}L_{n3}^{2}+M_{n3}L_{n2}L_{n3}\cos\left(\varphi_{3}\right),$$ (21)

$$Q_{31}=M_{n3}L_{n3}^{2}+M_{n3}L_{n1}L_{n3}\cos\left(\varphi_{2}+\varphi_{3}\right)+M_{n3}L_{n2}L_{n3}\cos\left(\varphi_{3}\right),$$ (22)

$$Q_{32}=M_{n3}L_{n3}^{2}+M_{n3}L_{n2}L_{n3}\cos\left(\varphi_{3}\right),$$ (23)

$$Q_{33}=M_{n3}L_{n3}^{2}.$$ (24)

The centrifugal term ( Pc ) is defined as illustrated in Equations 25–28:

$$P_{c}=\left[\begin{array}{c} P_{c1}\\ P_{c2}\\ P_{c3} \end{array}\right],$$ (25)

$$\begin{array}{ll} P_{c1} & =-L_{n1}\left(M_{n3}L_{n3}\sin\left(\varphi_{2}+\varphi_{3}\right)+M_{n2}L_{n2}\sin\left(\varphi_{2}\right)+M_{n3}L_{n2}\sin\left(\varphi_{2}\right)\right)\\ & \;\times \;{\dot{\varphi }}_{2}^{2}\;-M_{n3}L_{n3}\left(L_{n1}\sin\left(\varphi_{2}+\varphi_{3}\right)+L_{n2}\sin\left(\varphi_{3}\right)\right){\dot{\varphi }}_{3}^{2,} \end{array}$$ (26)

$$\begin{array}{ll} P_{c2} & =L_{n1}\left(M_{n3}L_{n3}\sin\left(\varphi_{2}+\varphi_{3}\right)+M_{n2}L_{n2}\sin\left(\varphi_{2}\right)+M_{n3}L_{n2}\sin\left(\varphi_{2}\right)\right)\\ & \;\times \;{\dot{\varphi }}_{1}^{2}-M_{n3}L_{n2}L_{n3}\sin\left(\varphi_{3}\right){\dot{\varphi }}_{3,}^{2} \end{array}$$ (27)

$$P_{c3}=M_{n3}L_{n3}\left(L_{n1}\sin\left(\varphi_{2}+\varphi_{3}\right)+L_{n2}\sin\left(\varphi_{3}\right)\right){\dot{\varphi }}_{1}^{2}+M_{n3}L_{n2}L_{n3}\sin\left(\varphi_{3}\right){\dot{\varphi }}_{2.}^{2}$$ (28)

The Coriolis term R is defined as presented in Equations 29–32:

$$R=\left[\begin{array}{c} R_{1}\\ R_{2}\\ R_{3} \end{array}\right],$$ (29)

$$\begin{array}{ll} R_{1} & =-2L_{n1}\left(M_{n3}L_{n3}\sin\left(\varphi_{2}+\varphi_{3}\right)+\left(M_{n2}+M_{n3}\right)L_{n2}\sin\left(\varphi_{2}\right)\right)\\ & \;\times \;{\dot{\varphi }}_{1}{\dot{\varphi }}_{2}-2M_{n3}L_{n3}\left(L_{n1}\sin\left(\varphi_{2}+\varphi_{3}\right)+L_{n2}\sin\left(\varphi_{3}\right)\right){\dot{\varphi }}_{2}{\dot{\varphi }}_{3},\\ & \;-2M_{n3}L_{n3}\left(L_{n1}\sin\left(\varphi_{2}+\varphi_{3}\right)+L_{n2}\sin\left(\varphi_{3}\right)\right){\dot{\varphi }}_{1}{\dot{\varphi }}_{3} \end{array}$$ (30)

$$R_{2}=-2M_{n3}L_{n2}L_{n3}\sin\left(\varphi_{3}\right){\dot{\varphi }}_{1}{\dot{\varphi }}_{3}-2M_{n3}L_{n2}L_{n3}\sin\left(\varphi_{3}\right){\dot{\varphi }}_{2}{\dot{\varphi }}_{3,}$$ (31)

$$R_{3}=2M_{n3}l_{n2}l_{n3}\sin\left(\varphi_{3}\right){\dot{\varphi }}_{1}{\dot{\varphi }}_{2}.$$ (32)

The potential energy term, G , is defined in Equations 33–36:

$$G=\left[\begin{array}{c} G_{1}\\ G_{2}\\ G_{3} \end{array}\right],$$ (33)

$$\begin{array}{ll} G_{1} & =\left(M_{n1}+M_{n2}+M_{n3}\right)gL_{n1}\cos\left(\varphi_{1}\right)+\left(M_{n2}+M_{n3}\right)gL_{n2}\\ & \;\times \;\cos\left(\varphi_{1}+\varphi_{2}\right)+M_{n3}gL_{n3}\cos\left(\varphi_{1}+\varphi_{2}+\varphi_{3}\right), \end{array}$$ (34)

$$G_{2}=\left(M_{n2}+M_{n3}\right)gL_{n2}\cos\left(\varphi_{1}+\varphi_{2}\right)+M_{n3}gL_{n3}\cos\left(\varphi_{1}+\varphi_{2}+\varphi_{3}\right),$$ (35)

$$G_{3}=M_{n3}gL_{n3}\cos\left(\varphi_{1}+\varphi_{2}+\varphi_{3}\right).$$ (36)

Using forward kinematic (Kumar et al., 2019), the joint angles $\varphi_{r1}$ , $\varphi_{r2},\text{and }\varphi_{r3}$ provide the coordinates for 3-LRRM’s end effector, as indicated by Equations 37, 38:

Regarding the reference trajectory,

$$x_{r}=L_{n1}\cos\left(\varphi_{r1}\right)+L_{n2}\cos\left(\varphi_{r1}+\varphi_{r2}\right)+L_{n3}\cos\left(\varphi_{r1}+\varphi_{r2}+\varphi_{r3}\right),$$ (37)

$$y_{r}=L_{n1}\sin\left(\varphi_{r1}\right)+L_{n2}\sin\left(\varphi_{r1}+\varphi_{r2}\right)+L_{n3}\sin\left(\varphi_{r1}+\varphi_{r2}+\varphi_{r3}\right).$$ (38)

where $\varphi_{r1}$ , $\varphi_{r2}\;and\;\varphi_{r3}$ are the desired trajectories.

Table 1 (Mohamed et al., 2023) provides a detailed description of the robot parameter settings used in this work.

**TABLE 1:** Nominal values of 3-LRRM parameters.

Parameter name	Nominal parameter value
$L_{n1}$	0.8 M
$L_{n2}$	0.4 M
$L_{n3}$	0.2 M
$M_{n1}$	0.1 Kg
$M_{n2}$	0.1 Kg
$M_{n3}$	0.1 Kg
g	9.81 M/ $S^{2}$

## 3 Proposed controller structures

**FIGURE 2:**
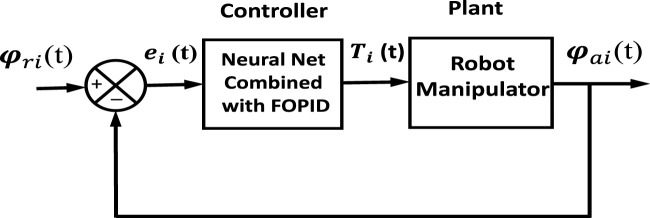
Schematic diagram of combined NN with FOPID controller.

Detailed information regarding the proposed hybrid neural network controllers’ structures is presented in this section. The closed-loop block diagram of the NN-like FOPID is given in Figure 2, where $\varphi_{ri}\left(t\right)$ is the desired position, $\varphi_{ai\;}\left(t\right)$ is the actual position, $e_{i}\left(t\right)$ is the error position, and $\varphi_{ri}\left(t\right)$ is the control signal.

### 3.1 Neural network-like FOPI and FOPD controller

**FIGURE 3:**
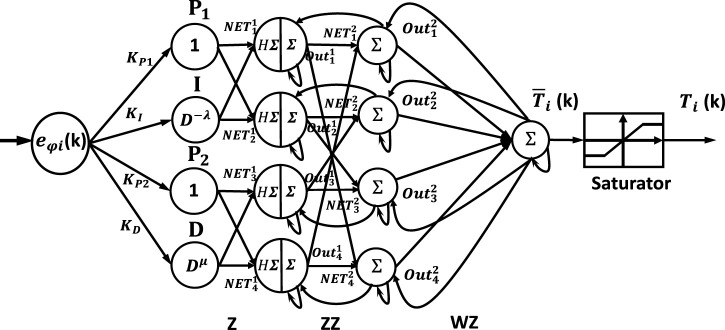
Neural controller PIPD controller.

The fundamental structure of the NN-FOPIPD controller is demonstrated in Figure 3, where the variables λ and μ are fractional values in the range of 0–2.

The difference between the desired position $\varphi_{ri}\left(t\right)$ and actual position $\varphi_{ai}\left(t\right)$ of the i link is known as the error ${\;e}_{\varphi i}\left(t\right)$ . The equations describing the controller from the error signal ${\;e}_{\varphi i}\left(t\right)$ to the control signal $\bar{T_{i}}\left(k\right)$ are shown below in Equations 39–48.

The single-processing element input layer is represented as ${\;e}_{\varphi i}\left(t\right)$ .

The first hidden layer has four processing elements: proportional operation $P_{1\;}$ , fractional integral operation I, proportional operation $P_{2}$ , and fractional derivative operation D, as follows:

$$P_{1}\left(t\right)=K_{P1}e_{\varphi i}\left(t\right)\;\text{or}\;P_{1}\left(k\right)=K_{P1}e_{\varphi i}\left(k\right),$$ (39)

$$I\left(t\right)=K_{I\;}D^{-\lambda }\;e_{\varphi i}\left(t\right)\;\text{ or }\;I\left(k\right)=K_{I\;}D^{-\lambda }\;e_{\varphi i}\left(k\right),$$ (40)

$$P_{2}\left(t\right)=K_{P2}{\;e}_{\varphi i}\left(t\right)\;\text{ or }\;P_{2}\left(k\right)=K_{P2}{\;e}_{\varphi i}\left(k\right),$$ (41)

$$D\left(t\right)=K_{D\;}D^{\mu }\;e_{\varphi i}\left(t\right)\;\text{ or }\;D\left(k\right)=K_{D\;}D^{\mu }\;e_{\varphi i}\left(k\right),$$ (42)

Where:

$$\left[\begin{array}{c} {NET}_{1}^{1}\left(k\right)\\ {NET}_{2}^{1}\left(k\right)\\ {NET}_{3}^{1}\left(k\right)\\ {NET}_{4}^{1}\left(k\right) \end{array}\right]=\left[\begin{array}{cccc} z11 & z12 & 0 & 0\\ \;z21\; & z22 & \;0\; & 0\\ 0 & 0 & z33 & z34\\ 0 & 0 & z43 & z44 \end{array}\right]\left[\begin{array}{c} P_{1}\left(k\right)\\ I\left(k\right)\\ P_{2}\left(k\right)\\ D\left(k\right) \end{array}\right],$$ (43)

$$\left[\begin{array}{c} {Out}_{1}^{1}\left(k\right)\\ {Out}_{2}^{1}\left(k\right)\\ {\;Out}_{3}^{1}\left(k\right)\\ {\;Out}_{4}^{1}\left(k\right) \end{array}\right]=\left[\begin{array}{c} H{\left(NET\right.}_{1}^{1}\left(k\right))\\ H{\left(NET\right.}_{2}^{1}\left(k\right))\\ H\left({NET}_{3}^{1}\left(k\right)\right)\\ H\left({NET}_{4}^{1}\left(k\right)\right) \end{array}\right]+\left[\begin{array}{c} z13\times {Out}_{1}^{1}\left(k-1\right)\\ z14\times {Out}_{2}^{1}\left(k-1\right)\\ z23\times {Out}_{3}^{1}\left(k-1\right)\\ z24\times {Out}_{4}^{1}\left(k-1\right) \end{array}\right]+\left[\begin{array}{c} z31\times {Out}_{1}^{2}\left(k-1\right)\\ z32\times {Out}_{2}^{2}\left(k-1\right)\\ z41\times {Out}_{3}^{2}\left(k-1\right)\\ z42\times {Out}_{4}^{2}\left(k-1\right) \end{array}\right],$$ (44)

Where:

$$H=\frac{4}{\left(1+e^{-net}\right)}-2,$$ (45)

$$\left[\begin{array}{c} {Out}_{1}^{2}\left(k\right)\\ {Out}_{4}^{2}\left(k\right) \end{array}\right]=\left[\begin{array}{c} zz11\;zz12\\ zz21\;zz22 \end{array}\right]\left[\begin{array}{c} {Out}_{1}^{1}\left(k\right)\\ {Out}_{4}^{1}\left(k\right) \end{array}\right]+\left[\begin{array}{c} {zz13\times Out}_{1}^{2}\left(k-1\right)\\ zz23{\times Out}_{4}^{2}\left(k-1\right) \end{array}\right]+\left[\begin{array}{c} zz14\times \bar{T}\left(k-1\right)\\ zz24\times \bar{T}\left(k-1\right) \end{array}\right],$$ (46)

$$\left[\begin{array}{c} {Out}_{2}^{2}\left(k\right)\\ {Out}_{3}^{2}\left(k\right) \end{array}\right]=\left[\begin{array}{c} zz31\;zz32\\ zz41\;zz42 \end{array}\right]\left[\begin{array}{c} {Out}_{2}^{1}\left(k\right)\\ {Out}_{3}^{1}\left(k\right) \end{array}\right]+\left[\begin{array}{c} {zz33\times Out}_{2}^{2}\left(k-1\right)\\ zz43{\;\times \;Out}_{3}^{2}\left(k-1\right) \end{array}\right]+\left[\begin{array}{c} zz34\times \bar{T}\left(k-1\right)\\ zz44\times \bar{T}\left(k-1\right) \end{array}\right],$$ (47)

$$\begin{array}{ll} \bar{T_{i}}\left(k\right) & =\bar{T_{i}}\left(k-1\right)+{wz}_{1}\;\times \;{Out}_{1}^{2}\left(k\right)+{wz}_{2}{\;\times \;Out}_{2}^{2}\left(k\right)+{wz}_{3}\\ & \;\times {Out}_{3}^{2}\left(k\right)+{wz}_{4}\;{\;\times \;Out}_{4}^{2}\left(k\right), \end{array}$$ (48)

### 3.2 Design of NN+FOPID

**FIGURE 4:**
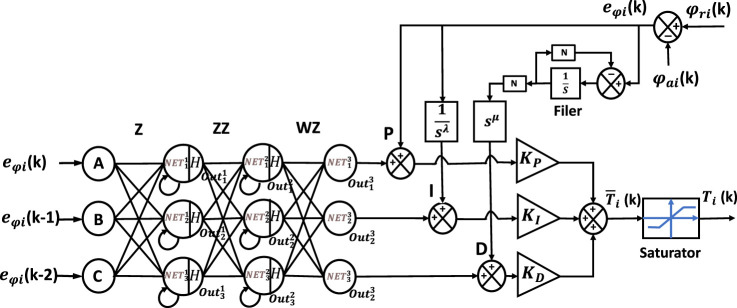
Neural network plus FOPID controller.

This controller contains two parts, as shown in Figure 4. The first part is the neural network; the input layer has three input elements, ${\;e}_{\varphi i}\left(k\right),e_{\varphi i}\left(k-1\right)$ and $e_{\varphi i}\left(k-2\right)$ or A, B, and C elements. The second part of the controller is the FOPID controller with a filter; both are merged and connected with the system in cascade.

The equations describing the controller from the error signal ${\;e}_{\varphi i}\left(t\right)$ to control signal $\bar{T_{i}}\left(k\right)$ are shown below in Equations 49–62.

Where:

$$\left[\begin{array}{c} {NET}_{1}^{1}\left(k\right)\\ {NET}_{2}^{1}\left(k\right)\\ {NET}_{3}^{1}\left(k\right)\; \end{array}\right]=\left[\begin{array}{ccc} z11 & z12 & z13\\ z21 & z22 & z23\\ z31 & z32 & z33 \end{array}\right]\left[\begin{array}{c} e_{\varphi i}\left(k\right)\\ e_{\varphi i}\left(k-1\right)\\ e_{\varphi i}\left(k-2\right) \end{array}\right]+\left[\begin{array}{c} {NET}_{1}^{1}\left(k-1\right)\\ {NET}_{2}^{1}\left(k-1\right)\\ {NET}_{3}^{1}\left(k-1\right)\; \end{array}\right],$$ (49)

The first hidden layer’s output is defined in Equation 50:

$$\left[\begin{array}{c} {Out}_{1}^{1}\left(k\right)\\ {Out}_{2}^{1}\left(k\right)\\ {Out}_{3}^{1}\left(k\right)\; \end{array}\right]=\left[\begin{array}{c} H\left({NET}_{1}^{1}\left(k\right)\right)\\ {H(NET}_{2}^{1}\left(k\right))\\ H\left({NET}_{3}^{1}\left(k\right)\right)\; \end{array}\right],$$ (50)

$$\left[\begin{array}{c} {NET}_{1}^{2}\left(k\right)\\ {NET}_{2}^{2}\left(k\right)\\ {NET}_{3}^{2}\left(k\right)\; \end{array}\right]=\left[\begin{array}{ccc} zz11 & zz12 & zz13\\ \;zz21\; & zz22 & \;zz23\;\\ zz31 & zz32 & zz33 \end{array}\right]\left[\begin{array}{c} {Out}_{1}^{1}\left(k\right)\\ {Out}_{2}^{1}\left(k\right)\\ {Out}_{3}^{1}\left(k\right)\; \end{array}\right]et+\left[\begin{array}{c} {NET}_{1}^{2}\left(k-1\right)\\ {NET}_{2}^{2}\left(k-1\right)\\ {NET}_{3}^{2}\left(k-1\right)\; \end{array}\right].$$ (51)

The second hidden layer’s output is indicated in Equation 52:

$$\left[\begin{array}{c} {Out}_{1}^{2}\left(k\right)\\ {Out}_{2}^{2}\left(k\right)\\ {Out}_{3}^{2}\left(k\right)\; \end{array}\right]=\left[\begin{array}{c} {H(NET}_{1}^{2}\left(k\right))\\ H\left({NET}_{2}^{2}\left(k\right)\right)\\ H\left({NET}_{3}^{2}\left(k\right)\right) \end{array}\right].$$ (52)

As can be noticed in Equation 52, the activation function is a sigmoid.

$$H=\frac{2}{\left(1+e^{-net}\right)}-1.$$ (53)

Equation 54 displays the output of the third and last hidden layer.

$$\left[\begin{array}{c} {Out}_{1}^{3}\left(k\right)\\ {Out}_{2}^{3}\left(k\right)\\ {Out}_{3}^{3}\left(k\right)\; \end{array}\right]=\left[\begin{array}{c} {NET}_{1}^{3}\left(k\right)\\ {NET}_{2}^{3}\left(k\right)\\ {NET}_{3}^{3}\left(k\right)\; \end{array}\right]=\left[\begin{array}{ccc} wz11 & wz12 & wz13\\ wz21 & wz22 & wz23\\ wz31 & wz32 & wz33 \end{array}\right]\left[\begin{array}{c} {Out}_{1}^{2}\left(k\right)\\ {Out}_{2}^{2}\left(k\right)\\ {Out}_{3}^{2}\left(k\right)\; \end{array}\right].$$ (54)

The three FOPID control operations are displayed in Equations 55–61.

$$P\left(t\right)=e_{\varphi i}\left(t\right)\;\text{ or }\;P\left(k\right)=e_{\varphi i}\left(k\right).$$ (55)

$$I\left(t\right)=D^{-\lambda }\;e_{\varphi i}\left(t\right)\text{ or }\;I\left(k\right)=D^{-\lambda }\;e_{\varphi i}\left(k\right).$$ (56)

$$f_{\varphi i}\left(s\right)=\frac{N}{s+N}e_{\varphi i}\left(s\right);\text{ The filter of derivative}.$$ (57)

$$D\left(t\right)=D^{\mu }\;f_{\varphi i}\left(t\right)\text{ or }D\left(k\right)=D^{\mu }\;f_{\varphi i}\left(k\right).$$ (58)

$${u_{1}\left(k\right)=K}_{P}\;\left({Out}_{1}^{3}\left(k\right)+P\left(k\right)\right).$$ (59)

$${u_{2}\left(k\right)=K}_{I}\;\left({Out}_{2}^{3}\left(k\right)+I\left(k\right)\right).$$ (60)

$${u_{3}\left(k\right)=K}_{D}\;\left({Out}_{3}^{3}\left(k\right)+D\left(k\right)\right).$$ (61)

Equation 62 illustrates the control signal equation.

$${\bar{T}}_{i}\left(k\right)=u_{1}\;\left(k\right)+u_{2}\;\left(k\right)+u_{3}\;\left(k\right).$$ (62)

### 3.3 Design of the Elman NN-FOPID

**FIGURE 5:**
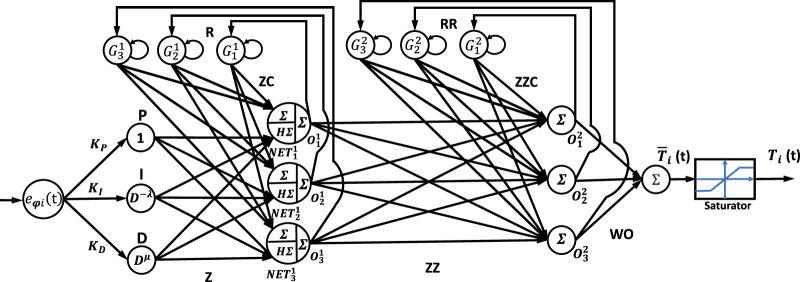
The structure of the Elman neural network-like FOPID controller.

The Elman neural network FOPID controller’s structural layout is depicted in Figure 5.

The difference between a required position $\varphi_{ri}\left(t\right)$ and an actual position $\varphi_{ai}\left(t\right)$ of the i link is known as the error $e_{\varphi i}\left(t\right)$ . The equations describing the controller from the error signal $e_{\varphi i}\left(t\right)$ to control signal $\bar{T_{i}}\left(k\right)$ are shown below in Equations 63–72.

The representation of the input layer with a single input element is ${\;e}_{\varphi i}\left(t\right)$ .

The first hidden layer has three processing elements (proportional operation P, integral operation I, and derivative operation D); the input to each node is the error $e_{\varphi i}\left(t\right)$ multiplied by a certain gain, while the activation functions represent one of the operations P, I, and D, and the output of each node is the result of its operation as described in Equations 63–65:

$$P\left(t\right)=K_{P}e_{\varphi i}\left(t\right)\;\text{ or }\;P\left(k\right)=K_{P}e_{\varphi i}\left(k\right),$$ (63)

$$I\left(t\right)=K_{I\;}D^{-\lambda }\;e_{\varphi i}\left(t\right)\;\text{ or }\;I\left(k\right)=K_{I\;}D^{-\lambda }\;e_{\varphi i}\left(k\right),$$ (64)

$$D\left(t\right)=K_{D\;}D^{\mu }\;e_{\varphi i}\left(t\right)\;\text{ or }\;D\left(k\right)=K_{D\;}D^{\mu }\;e_{\varphi i}\left(k\right),$$ (65)

$$\left[\begin{array}{c} {NET}_{1}^{1}\left(k\right)\\ {NET}_{2}^{1}\left(k\right)\\ {NET}_{3}^{1}\left(k\right)\; \end{array}\right]=\left[\begin{array}{c} \sum_{1}^{1}\left(k\right)\\ \sum_{2}^{1}\left(k\right)\\ \sum_{3}^{1}\left(k\right)\; \end{array}\right]=\left[\begin{array}{ccc} z11 & z12 & z13\\ \;z21\; & z22 & \;z23\;\\ z31 & z32 & z33 \end{array}\right]\left[\begin{array}{c} P\left(k\right)\\ I\left(k\right)\\ D\left(k\right) \end{array}\right],$$ (66)

and

$$\left[\begin{array}{c} G_{1}^{1}\left(k\right)\\ G_{2}^{1}\left(k\right)\\ G_{3}^{1}\left(k\right)\; \end{array}\right]=\left[\begin{array}{c} O_{1}^{1}\left(k-1\right){\;+\;r1\times G}_{1}^{1}\left(k-1\right)\\ O_{2}^{1}\left(k-1\right){\;+\;r2\times G}_{2}^{1}\left(k-1\right)\\ O_{3}^{1}\left(k-1\right){\;+\;r3\times G}_{3}^{1}\left(k-1\right)\; \end{array}\right].$$ (67)

The second hidden layer’s output is indicated in Equation 68:

$$\left[\begin{array}{c} O_{1}^{1}\left(k\right)\\ O_{2}^{1}\left(k\right)\\ {\;O}_{3}^{1}\left(k\right)\; \end{array}\right]=\left[\begin{array}{c} H\left(\sum_{1}^{1}\left(k\right)\right)\\ H\left(\sum_{2}^{1}\left(k\right)\right)\\ H\left(\sum_{3}^{1}\left(k\right)\right) \end{array}\right]+\left[\begin{array}{ccc} zc11 & zc12 & zc13\\ \;zc21\; & zc22 & \;zc23\;\\ zc31 & zc32 & vc33 \end{array}\right]\left[\begin{array}{c} G_{1}^{1}\left(k\right)\\ G_{2}^{1}\left(k\right)\\ G_{3}^{1}\left(k\right)\; \end{array}\right].$$ (68)

According to Equation 69, the activation function is a sigmoid function.

$$H=\frac{2}{\left(1+e^{-net}\right)}-1.$$ (69)

$$\left[\begin{array}{c} G_{1}^{2}\left(k\right)\\ G_{2}^{2}\left(k\right)\\ G_{3}^{2}\left(k\right)\; \end{array}\right]=\left[\begin{array}{c} O_{1}^{2}\left(k-1\right){\;+\;rr1\times G}_{1}^{2}\left(k-1\right)\\ O_{2}^{2}\left(k-1\right){\;+\;rr2\times G}_{2}^{2}\left(k-1\right)\\ {\;O}_{3}^{2}\left(k-1\right){\;+\;rr3\times G}_{3}^{2}\left(k-1\right)\; \end{array}\right].$$ (70)

The output of the third hidden layer is displayed in Equation 71:

$$\left[\begin{array}{c} O_{1}^{2}\left(k\right)\\ O_{2}^{2}\left(k\right)\\ {\;O}_{3}^{2}\left(k\right)\; \end{array}\right]=\left[\begin{array}{ccc} zz11 & zz12 & zz13\\ \;zz21\; & zz22 & \;zz23\;\\ zz31 & zz32 & zz33 \end{array}\right]\left[\begin{array}{c} O_{1}^{1}\left(k\right)\\ O_{2}^{1}\left(k\right)\\ {\;O}_{3}^{1}\left(k\right)\; \end{array}\right]+\left[\begin{array}{ccc} zzc11 & zzc12 & zzc13\\ \;zzc21\; & zzc22 & \;zzc23\;\\ zzc31 & zzc32 & zzc33 \end{array}\right]\left[\begin{array}{c} G_{1}^{2}\left(k\right)\\ G_{2}^{2}\left(k\right)\\ G_{3}^{2}\left(k\right)\; \end{array}\right].$$ (71)

The output layer consists of a single node, as shown in Equation 72:

$$\bar{T_{i}}\left(k\right)={wo}_{1}\times O_{1}^{2}\left(k\right)+{wo}_{2}\times O_{2}^{2}\left(k\right)+{wo}_{3}\;{\;\times \;O}_{3}^{2}\left(k\right),$$ (72)

where $K_{P},K_{I},K_{D},zij,zcij,zzij,zzcij,{wo}_{i},r_{i},\text{and }rr_{i},$ all are design parameters.

## 4 Bat optimization algorithm

This algorithm is inspired by the diverse emission rates and pulse intensities displayed by microbats during echolocation (Yang and He, 2013). Three primary rules can be used to explain the bat optimization algorithm: The first rule is to use the echolocation phenomenon to find the best distance to the food. According to the second rule, the bats fly at random with a fixed frequency and velocity at a particular search space location. However, the bats’ loudness and wavelength can vary based on their overall distance from their current location and the distance from the food. Lastly, the bat algorithm’s third rule is to linearly reduce the bat loudness factor’s behavior (Nor’Azlan et al., 2018). The following is the BOA procedure (Sambariya and Paliwal, 2016):

Step 1 Set initial values of the algorithm, including the issue’s dimension (*dim*), the highest value of iterations (*Iter*), the size of the population (*N*), and the minimum and maximum frequencies (*F*_min_ and *F*_*max*_), respectively. Additional variables are the bats’ initial velocity (*v*), their loudness (*A*_*l*_), their pulse emission rate (*Υ*), and their initial pulse emission rate (*ro*).

Step 2 Equation 73 can be used to generate a random initial population of bats, which is a feasible solution for each bat position.

$$x_{ij}=x_{j}^{l}+r\;and\left(\;\right)*\left(x_{j}^{u}-x_{j}^{l}\right).$$ (73)

The dimensions’ upper and lower bounds, *j*, are represented by $x_{j}^{l}$ and $x_{j}^{u}$ , respectively, and *i* = 1, …, *N*, *j* = 1, …, *dim*,

Step 3 Calculate the cost function (*MSE*) for every bat using Equation 74, and save the results in the cost vector.

$$MSE\left(i\right)=\frac{1}{N}\sum_{i=1}^{N}{\left(R-Y\right)}^{2},$$ (74)

where the desired signal is represented by *R* and the signal produced by Y.

Step 4 Determine the bat with the smallest (*MSE*) value as the (*F*_min_) among all bats in the vector is the best bat position (*x*∗).

Step 5 As indicated in Equations 75–77, update the *it*™ bat’s frequency, velocity, and position.

$$F_{i}=F_{\min}+\left(F_{\max}-F_{\min}\right)*r\;and\left(\;\right),$$ (75)

$$v_{i}^{ii}=v_{i}^{ii-1}+\left(x_{i}^{ii-1}-x^{*}\right)*F_{i,}$$ (76)

$$x_{i}^{ii}=x_{i}^{ii-1}+v_{i}^{ii},$$ (77)

where *ii* = 1, …, *Iter*.

Step 6 When a random number is less than the *ith* bat’s pulse emission rate (*Υi*), a local search is conducted. A new solution is generated using a random walk for the bat in position *i* to improve the diversity of possible solutions, as indicated in Equation 78.

$$x_{i}^{new}=x^{*}+\epsilon *{\langle A_{i}\rangle }^{ii}.$$ (78)

At the *ii* iteration, the total bats’ average noise level is represented by ${\;<A_{i}>}^{ii}$ , and *ϵ* is a scaling factor that is generated at random within the range [−1, 1].

Step 7 Utilizing Equation 74, determine the cost function value (*MSEi*) for the *i*th bat and enter the result in the variable.

Step 8 If the *i*th bat’s loudness ( $A_{li}$ ) is greater than a randomly generated number, its updated value of the objective or cost function is less than its previous value. Subsequently, the loudness, pulse rate, and updated cost function are computed, as shown in Equations 79–81, respectively.

Costi=CostN, (79)

$$A_{li}=\alpha \;*\;A_{li},$$ (80)

$$\Upsilon i=ro\;*\;\left(1\;–\;\exp\;\left(‐\beta \;*\;ii\right)\right),$$ (81)

where [0, 1] is the range of constants *α* and *β*.

Step 9 Using Equations 82–83, the minimum frequency (*F*_min_) and best bat position (*x*∗) for the *i*th bat can be updated if its cost *CostNi* is smaller than *F*_min_.

$$x*=x_{ij,}$$ (82)

$$F_{\left(\min\right)}=costNi.$$ (83)

Step 10 Proceed to Step 11 if the maximum number of bats (*N*) has been attained. If not, proceed to Step 5.

Step 11 End the procedure if the largest number of iterations (*Iter*) has been achieved. If not, repeat steps 5 through 11.

## 5 Simulation results

The trajectory tracking performance of the 3-LRRM nominal model with the proposed controllers is shown below. The proposed controllers for the 3-LRRM tracking problem are implemented using the MATLAB code. The torque limits for each link are set at (−200 to 200) N-m, the simulation period is taken 10 s, and the simulation’s step size is 1 msec. Moreover, the 11th-order Oustaloup’s approximation (N = 5), having a range of frequencies of (0.001, 1000) rad/s, is used in the fraction operator design. Each link’s controller is tuned via its parameters to track the required link. The overall performance goodness of all links will determine to what extent the accuracy of the manipulator end effector can maintain the required path. The basic objective for each link controller is to follow the desired path for this link with reduced error and match the desired path more quickly. In this work, the sum of ITSE for each link is used as the classical objective function in tuning all proposed controllers. The objective function is stated in Equation 84:

$$\min\;\;J=\int \left(t\times {e_{1}\left(t\right)}^{2}+t\times {e_{2}\left(t\right)}^{2}+t\times {e_{3}\left(t\right)}^{2}\right)\;dt.$$  (84)

The suggested controllers’ parameters are adjusted using the BOA algorithm in order to minimize the ITSE for each link. The controller is assessed according to the total tracking error ITSE for the three links between the reference paths and the calculated paths. Two initial positions (−0.15, −0.85, −1.15), (0.15, −0.55, −0.85) rad for Phi-1, Phi-2, and Phi-3, respectively, are taken into consideration in simulation to strengthen the training process for all suggested controllers. The fitness of a candidate solution is determined by adding the ITSE from these two executions. The BOA algorithm setting is a population size of 200, and the maximum value of iterations is 1500. Each suggested controller’s performance is assessed using ITSE computation. The controller that produces the lowest amount of ITSE is the best. Before discussing the outcomes of each suggested controller, we observed through numerous tests that the resulting control signal was typically highly chattering and inapplicable. This indicates that the design is unreliable and that the controller generates a control signal that the actuator cannot apply. In fact, neural networks have a high capability to map complicated underlying data structures, which is one of their key advantages. This capability produces the most complex high-frequency control signals in neural network controller design (i.e., chattering phenomenon). A chattering signal is actually impractical to use. Consequently, to solve this issue, the objective function is changed as follows:

The classical objective function is to minimize the ITSE, as expressed Equations 85, 86:

$$t\times {e\left(t\right)}^{2}=t\times {e_{1}\left(t\right)}^{2}+t\times {e_{2}\left(t\right)}^{2}+t\times {e_{3}\left(t\right)}^{2},$$ (85)

$$\min\;\;J=\int t\times {e\left(t\right)}^{2}\;dt.$$ (86)

The new objective function is as follows as presented in Equation 87:

$$\min\;\;J=\int {t\times e\left(t\right)}^{2}\;dt+Co\times \sigma .$$ (87)

σ is a small number chosen as $10^{-8}$

Co is a count of sign alterations in the control signal’s slope.

In the competition between the candidate solutions, this modified objective function will eliminate the solution that exhibits a high chattering control signal.

The desired trajectories $\varphi_{r1},$ $\varphi_{r2}$ and $\varphi_{r3}$ for link 1, link 2, and link 3 are provided in Equations 88–90, respectively:

$$\varphi_{r1}=\sin\left(0.2\pi t\right).$$ (88)

$${\;\varphi }_{r2}=\sin\left(0.2\pi t-\frac{\pi }{4}\right).$$ (89)

$${\;\varphi }_{r3}=\sin\left(0.2\pi t-\pi /2\right).$$ (90)

where the initial condition for $\varphi_{r1}=0,\varphi_{r2}=-0.7,and\;\;\varphi_{r3}=-1$ rad, and *x* (0) = 1.0769, *y* (0) = −0.4580 m.

**FIGURE 6:**
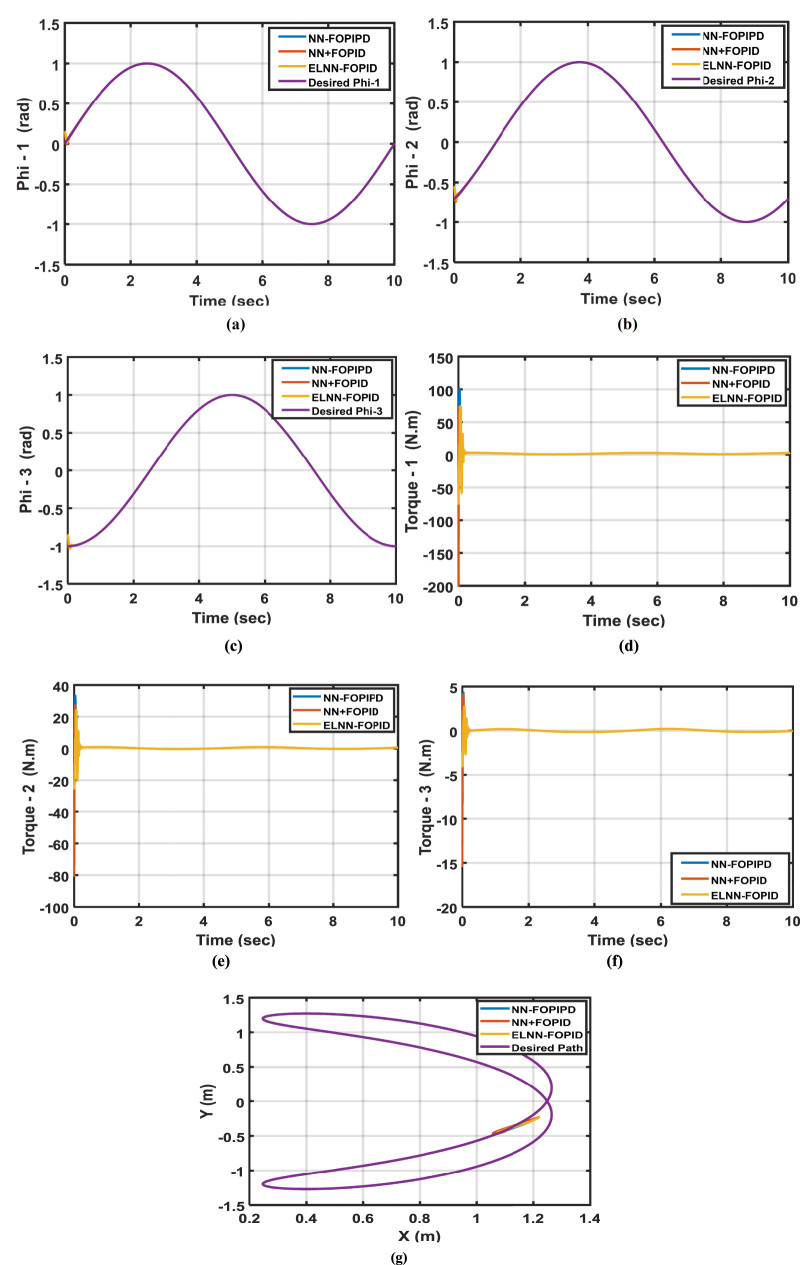
The desired and actual values for **(a)** Phi-1, **(b)** Phi-2, and **(c)** Phi-3. The control signals for **(d)** Control torque-1, **(e)** Control torque-2, **(f)** Control torque-3, and **(g)** the desired and actual end-effector paths.

Now, all of the nominal model and simulation data are available. First, to minimize the ITSE, we use the BOA to tune all the parameters of each proposed controller when using nominal model parameters. Because of the stochastic nature of the BOA, ten simulation runs were performed to get the best results for each controller simulation. Table 2 displays the performance index ITSE value for each suggested controller when a nominal plant is applied with two initial positions. Table 3 lists the features of each link’s control for all proposed controllers: settling time, rise time, maximum overshoot, and ITSE value when using the initial positions of 0.15 rad, −0.55 rad, and −0.85 rad. Figure 6 displays the trajectory tracking for each link of the proposed controller and its control signal, as well as end-effector x-y plots. Figures 6A–C show the desired and actual values for Phi-1, Phi-2, and Phi-3, respectively. Figures 6D,E show their control signals for Torque-1, Torque-2, and Torque-3, respectively, as well as the desired and actual path of the end effector in Figure 6G.

**TABLE 2:** ITSE and each control’s sign change at two initial positions (0.15, −0.55, −0.85) rad and (−0.15, −0.85, −1.15) rad when the nominal plant is applied.

Type of controller	ITSE	Number of slope sign Change in all control signals
NN-FOPIPD	**2.49695 × 10** ^**−5**^	**78**
NN + FOPID	**4.31033 × 10** ^**−5**^	**64**
ELNN-FOPID	**8.09808 × 10** ^**−5**^	**164**

**TABLE 3:** Specifications of the controller for each link when using a nominal system with starting positions (x1 = 0.15 rad, x2 = −0.55 rad, and x3 = −0.85 rad).

Controller Type	No of link	Rise Time	Settling Time	Overshoot %	ITSE ×10^−5^
NN-FOPIPD	L1	**0.040**	**0.060**	**5.778**	**0.34448**
	L2	**0.084**	**0.084**	**0.0014**	**0.41969**
	L3	**0.039**	**0.106**	**8.1747**	**0.42887**
NN + FOPID	L1	**0.039**	**0.457**	**32.889**	**1.50855**
	L2	**0.026**	**0.371**	**39.995**	**1.06976**
	L3	**0.026**	**0.093**	**18.209**	**0.24544**
ELNN-FOPID	L1	**0.052**	**0.219**	**21.086**	**0.93473**
	L2	**0.046**	**0.229**	**25.885**	**1.15868**
	L3	**0.053**	**0.366**	**21.697**	**1.49175**

From these results, it can be seen that the NN-FOPIPD controller structure gave smoother and faster convergence to the reference trajectory. In addition, the NN-FOPIPD gave the smallest overshoot, shortest settling time, and a closer trajectory to the reference trajectory with the lowest values of ITSE compared with the other controllers. The ELNN-FOPID controller yielded a value that was marginally higher than the NN-FOPIPD controller and relatively close to the NN-FOPIPD performance. The NN + FOPID controller has the lowest performance for each suggested controller. The NN-FOPIPD controller performs the best.

## 6 Robustness tests

In this section, each suggested controller’s ability, efficiency, and robustness will be demonstrated by evaluating its robustness without changing its parameters.

### 6.1 Change initial position

**FIGURE 7:**
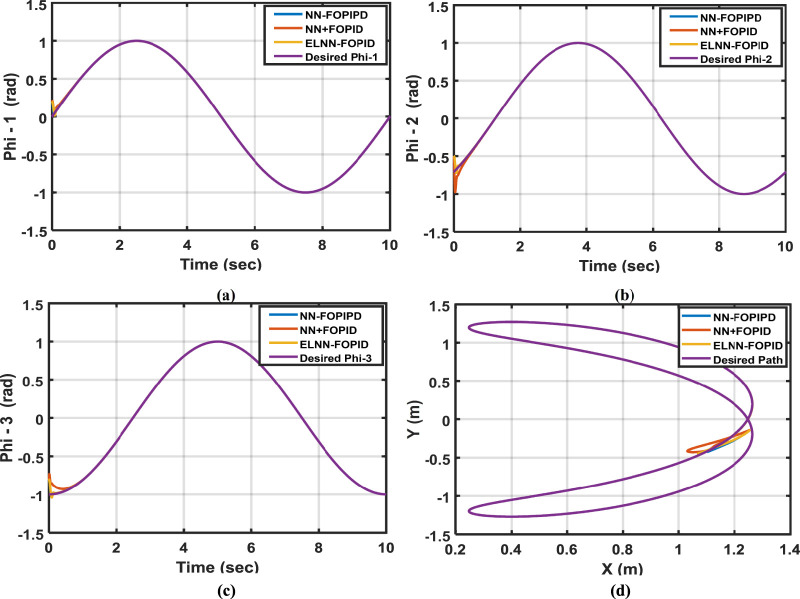
The desired and actual trajectories for **(a)** Phi-1, **(b)** Phi-2, and **(c)** Phi-3, respectively, and the **(d)** desired and actual end-effector paths when the initial positions of the links are (0.2, −0.5, −0.8) rad, respectively.

The robustness of the proposed controllers can be evaluated by altering the starting positions to (0.2, −0.5, and 0.8) rad for Phi-1, Phi-2, and Phi-3, respectively. In Figure 7, the trajectory of the 3-LRRM end effector is depicted when the initial positions of all links are changed, in addition to tracking Phi-1, Phi-2, and Phi-3 trajectories. The ITSE of the proposed control schemes is given in Table 4.

**TABLE 4:** ITSEs of the proposed schemes when the links’ initial values are (0.2, −0.5, −0.8) rad.

Controller	ITSE
NN-FOPIPD	**3.430154 × 10** ^**−5**^
NN + FOPID	**110.2063 × 10** ^**−5**^
ELNN-FOPID	**8.278723 × 10** ^**−5**^

As can be observed, the NN-FOPIPD controller has the smallest ITSE value, fastest response, and least settling time. Consequently, when Phi-1, Phi-2, and Phi-3’s starting positions are changed, the NN-FOPIPD controller performs better than the other suggested controllers.

### 6.2 Disturbance addition

**FIGURE 8:**
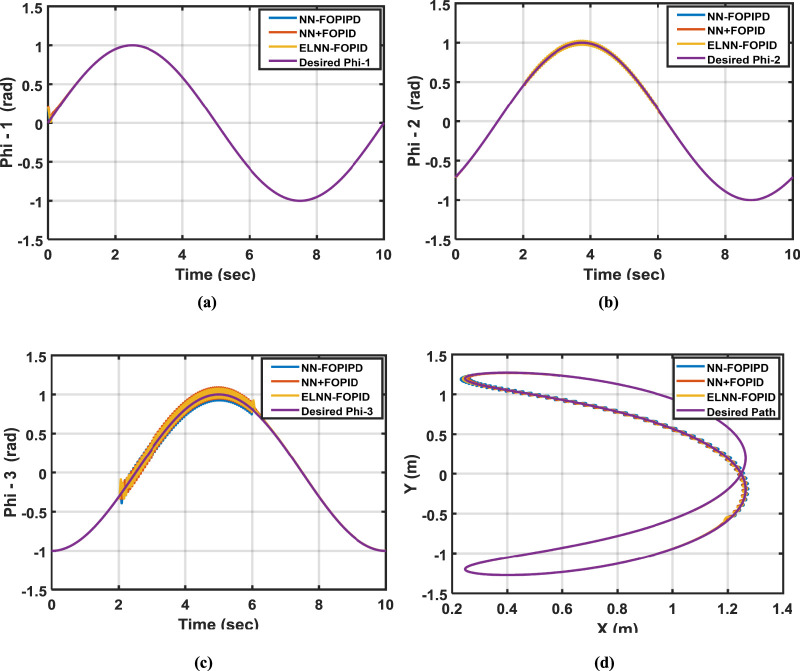
The desired and actual trajectories for **(a)** Phi-1, **(b)** Phi-2, and **(c)** Phi-3, respectively, and the **(d)** desired and calculated paths of the end effector when applying the external disturbance sin (100t) N-M to all link controllers while the starting position is (0, −0.7, −1) rad.

The capability of disturbance rejection for each suggested controller has been analyzed. This test is implemented after including disturbance term sin (100t) N-m for the duration of 2–6 s in the controller output of each link. In this test, the starting position is taken to be (0, −0.7, −1) rad. Table 5 displays the achieved ITSE results for each proposed controller. Figure 8 shows how the actual trajectory of the 3-LRRM end effector tracks the desired trajectory; in addition, it shows the trajectory tracking of Phi-1, Phi-2, and Phi-3 when the external disturbance is considered is sin (100t) N-m in each link.

**TABLE 5:** ITSE of the proposed scheme with starting conditions of 0 rad, −0.7 rad, and 1 rad, and disturbances added to all control signals in the period 2–6 s sin (100t).

Control scheme	ITSE
NN-FOPIPD	**0.062988**
NN + FOPID	**0.065961**
ELNN-FOPID	**0.050069**

The results clearly show that the suggested NN-FOPIPD and ELNN-FOPID controllers perform nearly equally well. However, the ELNN-FOPID gives the lowest value of ITSE and a slightly better performance than the NN-FOPIPD. The NN + FOPID controller gives a worse performance. Therefore, the ELNN-FOPID outperforms the other proposed controllers for disturbance rejection.

### 6.3 Parameter variations

**FIGURE 9:**
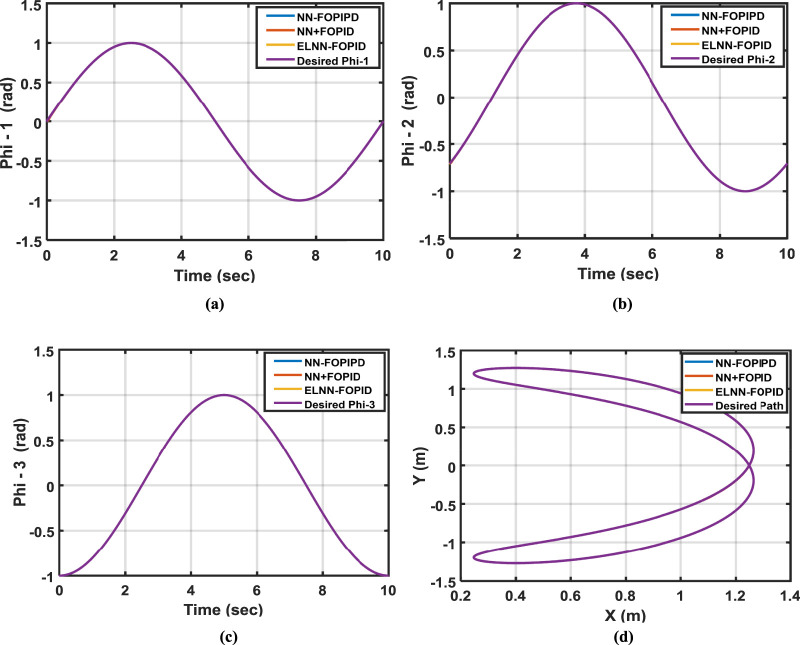
**(a)** The desired and calculated Phi-1, **(b)** Phi-2, **(c)** Phi-3, and **(d)** the desired and calculated paths of the end effector when 10% is added to the mass of link3 with starting positions for all links are (0, −0.7, −1) rad.

In industry, a manipulator’s main job is picking and placing different components with varying weights utilizing its end effector. In order to conduct this test, link 3’s masses will be increased by 10%, and the starting position (0.0, −0.7, −1) rad will be used. This test explores the proposed controller’s performance when parameter variation occurs in the system. Table 6 lists the calculated ITSEs for each suggested controller. The desired and actual trajectory tracking of Phi-1, Phi-2, and Phi-3 in relation to changing the weights for all controllers is displayed in Figure 9.

**TABLE 6:** The ITSE values for the proposed controllers when the mass of Link-3 is increased by 10% and the initial position is (0.0, −0.7, 1) rad.

Proposed schemes	ITSE
NN-FOPIPD	**0.00099 × 10** ^**−5**^
NN + FOPID	**0.30056 × 10** ^**−5**^
ELNN-FOPID	**0.00179 × 10** ^**−5**^

Based on the findings, the ITSE for the NN-FOPIPD controller is the lowest value among other controllers when parameter variation occurs. However, the NN-FOPIPD gave a fast response that was closest to the required trajectory for Phi-1, Phi-2, and Phi-3 when compared to other proposed controllers. Conversely, the highest (and worst) ITSE value was caused by applying the NN + FOPID controller.

### 6.4 All previous tests combined

**FIGURE 10:**
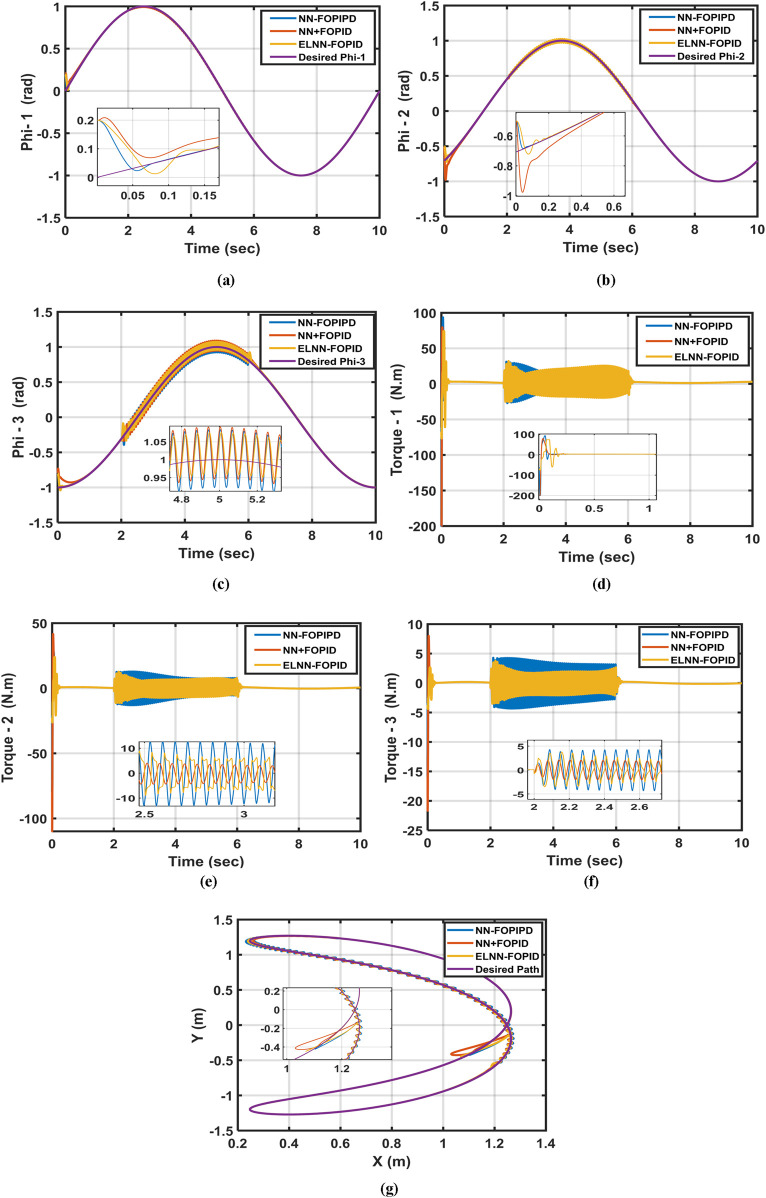
Desired and actual trajectories **(a)** Phi-1, **(b)** Phi-2, **(c)** Phi-3 as well as the corresponding control signals, **(d)** Control torque-1, **(e)** Control torque-2, **(f)** Control torque-3, and **(g)** actual path traced by the end effector when using starting position (0.2, −0.5, −0.8) rad, adding disturbance [sin (100t)] N-M for all links, and a 10% increase in link 3’s mass.

The efficiency of the suggested controllers is determined by combining all the effects on the control system. In this crucial test, all the proposed controllers are simultaneously subjected to the following effects: altering the initial position, adding a disturbance, and changing the parameters of the system model. Table 7 contains a list of the obtained ITSE values. Figure 10 shows the desired and actual Phi-1, Phi-2, and Phi-3 trajectories and the end-effector trajectories when the system is subjected to the above effects.

**TABLE 7:** The values of ITSE for all controllers when utilizing the initial position (0.2, −0.5, and −0.8) rad, adding external disturbance term sin (100t)*(1, 1, 1), and changing the third link mass by 10% simultaneously.

Control scheme	ITSE
NN-FOPIPD	**0.0630227**
NN + FOPID	**0.066909**
ELNN-FOPID	**0.050358**

Of the proposed controllers, the ELNN-FOPID has the lowest ITSE value even after changing the model parameters, adding a disturbance, and shifting the starting positions. The ELNN-FOPID controller demonstrates the fastest response, shortest settling times, closeness-to-required trajectories, and lowest energy consumption, as demonstrated by its Phi-1, Phi-2, and Phi-3 trajectories. The NN-FOPIPD controller comes in second order in performance and accuracy. On the other hand, the NN + FOPID controller has the worst performance because of its maximum settling time and highest ITSE value. We can conclude that the ELNN-FOPID controller is the best and outperforms in all states.

## 7 Conclusion

A robotic manipulator’s performance is negatively affected by parameter uncertainty and outside disturbances because it is a highly coupled, complex, nonlinear, and multi-input multi-output system. Consequently, the controllers designed for these systems must be able to handle their complexity. The goal of this work is to solve the nonlinear problems of compensating robot manipulator control with disturbances and uncertainties and achieving precise position by designing hybrid controllers that combine the advantages of neural network-based BOA and a PID controller utilizing BOA for 3-LRRM. The parameters of these controllers are adjusted in accordance with the definition of the objective function as the ITSE. All suggested controllers are for reference trajectory tracking when all effects are present, like altering initial positions, adding disturbance, and model parameter uncertainty. The simulation’s outcomes showed that the suggested NN-FOPIPD controller is most effective for tracking trajectories, rejecting disturbances, adjusting parameters, and having the lowest performance index ITSE value. In other words, the NN-FOPIPD controller exhibits superior tracking, stability, and robustness compared to the other suggested controllers. Future work and extension of the research idea can address the following reference ideas: Nam Dao et al. (2024), Nam Dao et al. (2025), Köhler et al. (2021), and Nguyen et al. (2024).

## Data Availability

The original contributions presented in the study are included in the article/supplementary material; further inquiries can be directed to the corresponding authors.
